# Circulating Syndecan-1 Levels Are Associated with Chronological Coagulofibrinolytic Responses and the Development of Disseminated Intravascular Coagulation (DIC) after Trauma: A Retrospective Observational Study

**DOI:** 10.3390/jcm12134386

**Published:** 2023-06-29

**Authors:** Hironori Matsumoto, Suguru Annen, Naoki Mukai, Muneaki Ohshita, Satoru Murata, Yutaka Harima, Shirou Ogawa, Mitsuo Okita, Yuki Nakabayashi, Satoshi Kikuchi, Jun Takeba, Norio Sato

**Affiliations:** Department of Emergency and Critical Care Medicine, Graduate School of Medicine, Ehime University, Toon 791-0295, Ehime, Japanyutaka13right@yahoo.co.jp (Y.H.); kiku@m.ehime-u.ac.jp (S.K.);

**Keywords:** trauma-induced coagulopathy (TIC), glycocalyx, endotheliopathy, coagulation, fibrinolysis

## Abstract

Background: The purpose of this study was to evaluate the association between endotheliopathy represented by high levels of circulating syndecan-1 (SDC-1) and coagulofibrinolytic responses due to trauma, which can lead to disseminated intravascular coagulation (DIC). Methods: We retrospectively evaluated 48 eligible trauma patients immediately admitted to our hospital and assessed SDC-1 and coagulofibrinolytic parameters for 7 days after admission. We compared the longitudinal changes of coagulofibrinolytic parameters and SDC-1 levels between two groups (high and low SDC-1) according to median SDC-1 value on admission. Results: The median circulating SDC-1 level was 99.6 (61.1–214.3) ng/mL on admission, and levels remained high until 7 days after admission. Coagulofibrinolytic responses assessed by biomarkers immediately after trauma were correlated with SDC-1 elevation (thrombin–antithrombin complex, TAT: *r* = 0.352, *p* = 0.001; antithrombin, AT: *r* = −0.301, *p* < 0.001; plasmin-α_2_-plasmin inhibitor complex, PIC: *r* = 0.503, *p* = 0.035; tissue plasminogen activator, tPA: *r* = 0.630, *p* < 0.001). Sustained SDC-1 elevation was associated with intense and prolonged coagulation activation, impairment of anticoagulation, and fibrinolytic activation followed by inhibition of fibrinolysis, which are the primary responses associated with development of DIC in the acute phase of trauma. Elevation of circulating SDC-1 level was also associated with consumption coagulopathy and the need for transfusion, which revealed a significant association between high SDC-1 levels and the development of DIC after trauma (area under the curve, AUC = 0.845, cut-off value = 130.38 ng/mL, *p* = 0.001). Conclusions: High circulating levels of syndecan-1 were associated with intense and prolonged coagulation activation, impairment of anticoagulation, fibrinolytic activation, and consumption coagulopathy after trauma. Endotheliopathy represented by SDC-1 elevation was associated with trauma induced coagulopathy, which can lead to the development of DIC.

## 1. Introduction

Trauma is one of the major causes of mortality accounting for approximately 10% of deaths worldwide [1]. The prevalence of coagulopathy immediately after trauma is approximately 25% to 30%, and early trauma deaths are primarily attributed to uncontrolled hemorrhage [2,3,4]. Trauma is known to induce dynamic coagulofibrinolytic responses, which increase the bleeding tendency in the initial phase of trauma when hemostasis becomes uncontrollable [2,3]. This trauma-induced coagulopathy (TIC) is caused by the trauma itself and exacerbated by multiple factors such as hemorrhage, shock, and hypothermia, which occasionally lead to fulminant disseminated intravascular coagulation (DIC) [3,5,6,7]. 

The endothelium plays a pivotal role in hemostasis and thrombosis. The endothelial layer is coated with endothelial cells and a layer called the glycocalyx. The glycocalyx is composed of membrane-bound proteoglycans, glycoproteins, glycosaminoglycans, and adherent plasma proteins [8,9,10,11]. Syndecan-1 (SDC-1) is a major cell surface heparan sulfate proteoglycan in the endothelial glycocalyx and maintains endothelial function by various processes such as modulating cell proliferation, leukocyte recruitment, angiogenesis, host defense, and matrix remodeling through biding to many mediators [9]. SDC-1 is shed into the blood when the endothelium is damaged, thus the circulating SDC-1 concentration may be a biomarker of endotheliopathy [8,9,10]. Trauma also causes endothelial damage and increases the permeability of the endothelium (endotheliopathy) [12,13]. Several studies have demonstrated that circulating SDC-1 concentrations increase after trauma and are associated with severity and mortality [14,15,16,17]. 

The pathogenesis of TIC is multifactorial, but some previous studies have suggested that endotheliopathy of trauma contributes to the development of coagulopathy [14,16,18]. However, the association of SDC-1 levels with comprehensive and chronological coagulofibrinolytic responses in the early phase of trauma has not been fully evaluated. TIC, which can lead to the development of DIC in the early phase of trauma, exhibits coagulation activation, impairment in anticoagulation, fibrinolytic activation complicated by delayed inhibition of fibrinolysis, and consumption coagulopathy [6,19,20]. By comprehensively analyzing these reactions, it becomes possible to gain a more detailed understanding of the pathophysiology of TIC. Furthermore, coagulofibrinolytic responses in trauma change drastically in the early phase; thus, a chronological assessment of changes in biomarkers of coagulofibrinolysis is essential [6,7,19]. By elucidating the relationship between coagulofibrinolytic responses following trauma and endotheliopathy as measured by SDC-1, we can potentially utilize SDC-1 as a valuable indicator of endotheliopathy associated with TIC. Furthermore, this association indicates the potential value in developing novel therapeutic strategies specifically targeting endotheliopathy in the management of TIC. The primary aim of our study was to investigate the relationship between circulating SDC-1 levels and comprehensive coagulofibrinolytic responses due to trauma by evaluating longitudinal data of coagulofibrinolytic markers. We conducted an initial investigation on the correlations between SDC-1 elevation and comprehensive coagulofibrinolytic responses immediately after trauma. Subsequently, we assessed the association between SDC-1 elevation and chronological changes in coagulofibrinolytic markers by comparing longitudinal data between patients with high and low SDC-1 concentrations based on the median SDC-1 concentration upon admission. Finally, we evaluated the relationship between SDC-1 elevation and clinical outcomes, including the development of DIC, the prevalence of shock, and in-hospital mortality.

## 2. Method

### 2.1. Study Design

We conducted a retrospective, single-center observational study of adult trauma patients admitted to the tertiary Ehime University Hospital in Japan from October 2017 to January 2022. The present study was conducted in accordance with the Declaration of Helsinki and was approved by the Ethics Committee for Clinical Research of Ehime University Hospital (No. 1902014). Informed consent was obtained from all patients or next of kin for research use of the leftover blood samples after routine blood tests in accordance with the Declaration of Helsinki.

### 2.2. Patient Selection and Criteria

All adult trauma patients (≥18 years) who were directly admitted to our hospital or were transferred from other hospitals without any significant therapeutic interventions were enrolled. Some therapeutic interventions such as radical hemostasis and antifibrinolytic therapy, and dilutional coagulopathy associated with massive fluid infusion, can strongly affect the chronological evaluation of the coagulofibrinolytic response after trauma; thus, we excluded patients who had already received therapeutic interventions, including infusion (more than 500 mL of fluid administration) or medications before transfer to our hospital. We also excluded patients who had died during initial treatment in the emergency department, those who had an episode of cardiac arrest, those who received anticoagulant and antiplatelet therapy, and those who had a clotting disorder such as liver cirrhosis or advanced malignancy. In accordance with hospital policy, we actively assessed coagulofibrinolytic parameters in the early phase of trauma, primarily immediately after admission, 3 h after admission, and on days 1, 2, 3, and 6. Serum samples for analyzing circulating SCD-1 values and with complete data of coagulofibrinolytic parameters for 7 days after admission were obtained from 48 patients. As a control group, we used data from laboratory tests of 10 healthy individuals with no known significant health problems.

Demographic data, examinations, treatments, and in-hospital mortality were recorded. Overt-DIC was diagnosed within 7 days after admission in accordance with the criteria established by the International Society on Thrombosis and Haemostasis (ISTH) [21,22]. Shock was diagnosed if the systolic blood pressure was sustained below 90 mmHg and lactate levels were > 18 mg/dL (approximately 2 mmol/L) in the emergency department. Transfusions of packed red blood cells (PRBC), fresh-frozen plasma (FFP), or platelets were allowed in order to maintain hemodynamics and hemostasis at the discretion of clinicians informed by the laboratory data. 

We stratified the patients into two groups according to the median SDC-1 concentration on admission to compare the coagulofibrinolytic responses between patients with high and low SDC-1 concentrations.

### 2.3. Blood Sampling and Measurement

Blood sampling was performed on admission (OA), 3 h after admission (3H), and on day (D)1, D2, D3, and D6. We routinely measured blood counts and biochemistries including albumin (Alb) using TBA-c16000 (Toshiba Medical Systems, Tochigi, Japan) and XE-5000 (Sysmex, Hyogo, Japan). We measured biomarkers of coagulofibrinolysis using CP-2000 (Sekisui Medical, Tokyo, Japan) and STACIA (LSI Medience, Tokyo, Japan), including prothrombin time (PT), activated partial thromboplastin time (APTT), fibrinogen (Fbg), fibrin/fibrinogen degradation product (FDP), D-dimer, soluble fibrin (SF), thrombin–antithrombin complex (TAT), plasmin-α_2_-plasmin inhibitor complex (PIC), antithrombin (AT), protein C (PC), α_2_-plasmin inhibitor (α_2_PI), and plasminogen (PLG). 

After sampling, the blood samples were centrifuged at 3300 rpm for 15 min at 4 °C, and serum and plasma samples were stored at −80 °C for subsequent analyses. We measured SDC-1, tissue plasminogen activator (tPA), and total plasminogen activator inhibitor-1 (tPAI-1) at OA, 3H, and D1, D3, and D6. SDC-1 and tPA were measured using an enzyme-linked immunosorbent assay (ELISA) (Human CD138 ELISA kit, Diaclone, Besancon, Cedex, France; Human t-Plasminogen Activator/tPA Immunoassay, R&D Systems, Minneapolis, MN, USA) according to the manufacturers’ instructions. tPAI-1 represents the sum of active PAI-1, t-PA/PAI-1, and latent PAI-1 and was measured by the LPIA·tPAI-1 test using the STACIA analyzer (LSI Medience Corporation, Tokyo, Japan). IL-6 was measured by ELISA (Human IL-6 Quantikine ELISA kit, R&D Systems, Minneapolis, MN, USA) on admission. tPAI-1 and IL-6 were measured at the central laboratory of the BML, Inc. (Tokyo, Japan). Serum samples were used for the measurement of SDC-1 and IL-6, and plasma samples were used for the measurement of tPA and tPAI-1. 

### 2.4. Statistical Analysis

Statistical analyses were performed using IBM SPSS statistics v. 22 (IBM, Tokyo, Japan) and R version 4.2.0 (https://cran.r-project.org/ accessed on 3 December 2022). All data are expressed as median (interquartile range). Comparisons between the two groups were performed using the Mann–Whitney *U* test, Student’s *t*-test, or Fisher exact test, as appropriate. Relationships between the dependent variables were analyzed using Pearson’s correlation coefficient analysis and a linear regression analysis with t-test. Differences between the longitudinal data of trauma patients and the data of healthy controls were compared with the Mann–Whitney *U* test. The longitudinal differences between groups were analyzed by two-way repeated-measures ANOVA, followed by Bonferroni’s post hoc test. Receiver operating characteristic (ROC) curves were used to determine the ability of SDC-1 levels to predict the development of DIC and shock. The cut-off values were also determined by the ROC analyses using the Youden index. The Kaplan–Meier analysis with log-rank test was used to estimate cumulative development of DIC. A *p* value less than 0.05 was considered significant.

## 3. Results

### 3.1. Patient Characteristics

The baseline characteristics of the 48 patients are shown in Table 1. The median age was 61.5 (42.0–72.6) years, the median injury severity score (ISS) was 19 (13–29), and 77.1% were men. The mechanism of trauma for all the patients was blunt injury: most patients had several organ injuries. Shock was observed in 13 patients (27.1%), and DIC developed in 11 patients (22.9%). DIC was diagnosed in two patients on admission, in seven patients within 3 h after admission, and in all cases within 24 h. A total of 23 (47.9%) and 31 (64.6%) patients, respectively, received transfusions and therapeutic interventions, including surgery and interventional radiology (IVR). The median duration from injury to blood sampling on admission was 60 (43–110) min. Parameters reflecting the systemic inflammatory response and coagulofibrinolytic activation notably increased on admission (IL-6, 122 [33–277] pg/mL; TAT, 115.3 [49.3–200.0] μg/L; PIC, 10.4 [4.8–27.8] μg/mL; tPA, 6468 [4208–10,411] pg/mL; FDP, 89.4 [27.3–164.3] μg/mL, D-dimer, 46.1 [14.7–82.5] μg/mL). Five patients (10.4%) had died, and four of them had died by 7 days after admission. No significant renal dysfunction was observed in any of the patients included in this study.

### 3.2. Circulating SDC-1 Changes after Trauma and Relationship between Circulating SDC-1 Levels and Coagulofibrinolytic Responses on Admission

**Figure 1:**
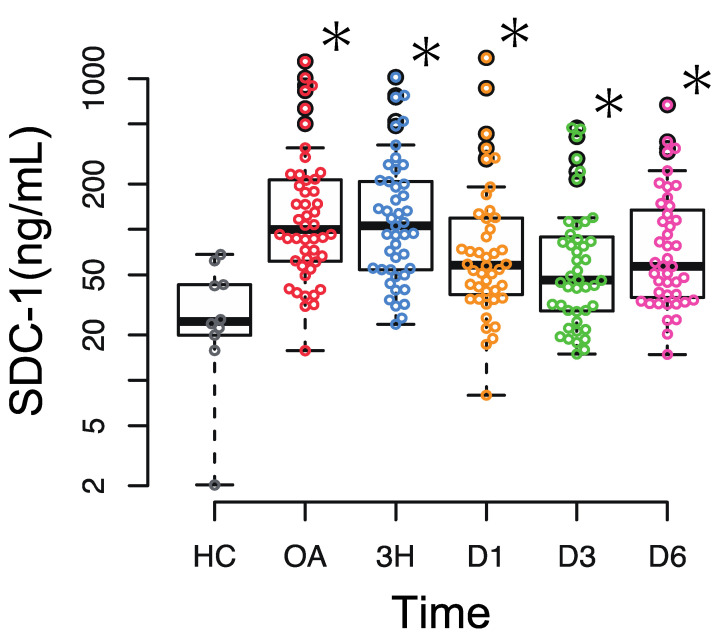
Circulating syndecan-1 (SDC-1) levels on admission (OA), 3 h after admission (3H), and on days 1, 3, and 6 (D1, D3, D6) compared with the levels of healthy control (HC). The central horizontal bars, columns, and peripheral longitudinal bars indicate the median values, 25th to 75th percentiles and 10th to 90th percentiles, respectively. * *p* < 0.05.

The median circulating SDC-1 level was 99.6 (61.1–214.3) ng/mL on admission (Table 1). SDC-1 increased immediately after injury, followed by a gradual decrease, but higher levels persisted in trauma patients than in healthy individuals until 7 days after admission (healthy individuals: 24.5 [18.9–47.8] ng/mL) (Figure 1). 

**Figure 2:**
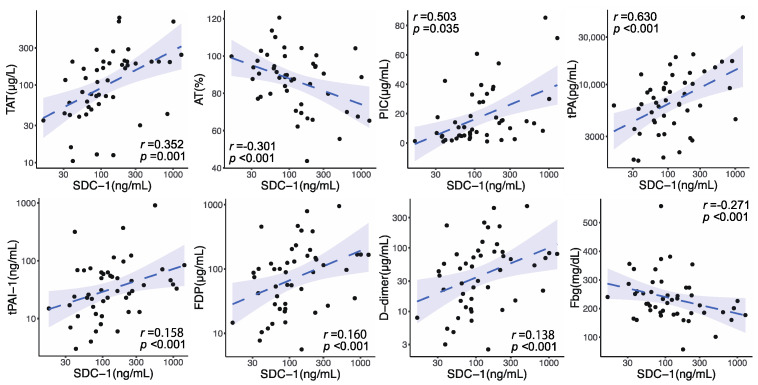
Correlations between syndecan-1 (SDC-1) and coagulofibrinolytic markers on admission. The dashed lines represent the regression lines, and the shaded areas represent 95% confidence intervals. *r*, correlation coefficient; *p*, *p* value. TAT, thrombin–antithrombin complex; AT, antithrombin; PIC, plasmin-α_2_-plasmin inhibitor complex; tPA, tissue plasminogen activator; tPAI-1, total plasminogen activator inhibitor-1; FDP, fibrin/fibrinogen degradation product; Fbg, fibrinogen.

Circulating SDC-1 values on admission were elevated in conjunction with many coagulofibrinolysis-related parameters (Supplementary Materials Figure S1). Coagulation activity represented by TAT, impairment in anticoagulation evaluated by AT activity, and fibrinolytic activity shown by PIC and tPA were significantly correlated with circulating SDC-1 levels on admission (TAT: *r* = 0.352, *p* = 0.001; AT: *r* = −0.301, *p* < 0.001; PIC: *r* = 0.503, *p* = 0.035; tPA: *r* = 0.630, *p* < 0.001) (Figure 2). A principal inhibitor of fibrinolysis, tPAI-1, was also significantly correlated with SDC-1 levels on admission (*r* = 0.158, *p* < 0.001). Coagulofibrinolytic responses indicated by FDP and D-dimer and consumption of Fbg also showed significant correlations with SDC-1 levels on admission (FDP: *r* = 0.160, *p* < 0.001; D-dimer: *r* = 0.138, *p* < 0.001; Fbg: *r* = −0.271, *p* < 0.001). 

### 3.3. Association between Circulating SDC-1 Elevation and Chronological Changes in Coagulofibrinolytic Markers

**Figure 3:**
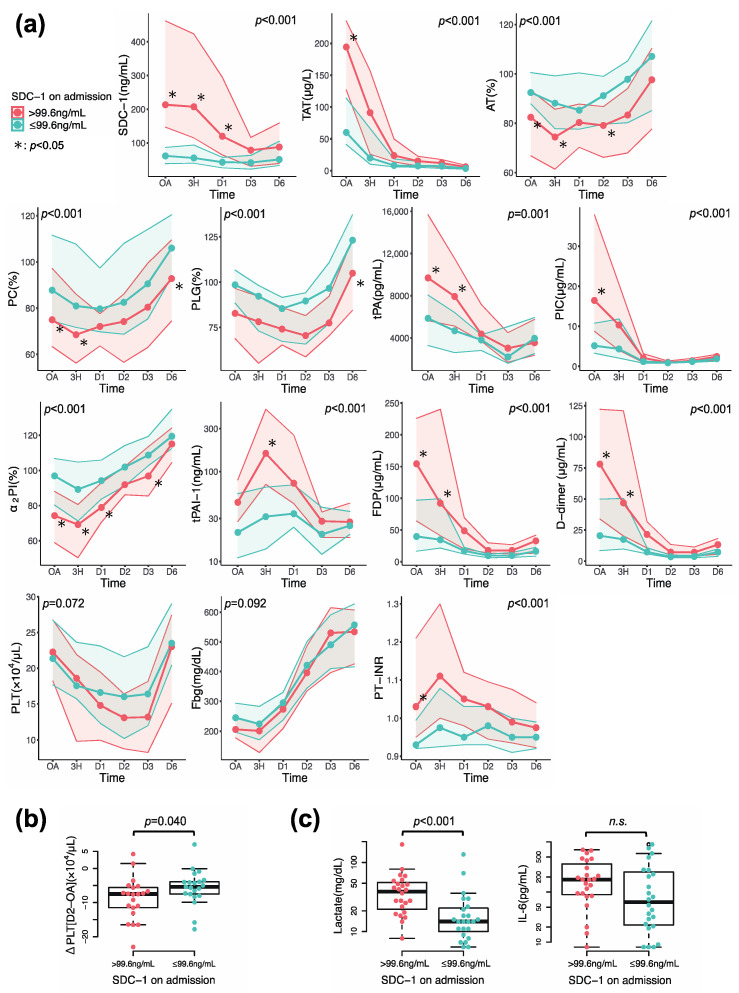
Comparisons of (**a**) time course changes in coagulofibrinolytic markers, (**b**) changes in PLT from OA to D2, and (**c**) lactate and IL-6 levels on admission based on the median syndecan-1 (SDC-1) level on admission. (**a**) The red lines represent the values of the patients with SDC-1 levels on admission > 99.6 ng/mL, and the green lines represent those with SDC-1 levels on admission ≤ 99.6 ng/mL. Central marks represent median values, and the shaded areas represent 25th to 75th percentiles. Statistical significance of differences was assessed by two-way repeated measures ANOVA (*p* value) with the Bonferroni post hoc test (* *p* < 0.05). TAT, thrombin–antithrombin complex; AT, antithrombin; PC, protein C; PLG, plasminogen; tPA, tissue plasminogen activator; PIC, plasmin-α_2_-plasmin inhibitor complex; α_2_PI, α_2_-plasmin inhibitor; tPAI-1, total plasminogen activator inhibitor-1; FDP, fibrin/fibrinogen degradation product; PLT, platelet; Fbg, fibrinogen; PT-INR, prothrombin time–international normalized ratio. (**b**,**c**) The red dots represent the values of the patients with SDC-1 levels on admission > 99.6 ng/mL, and the green dots represent those with SDC-1 levels on admission ≤ 99.6 ng/mL. The central horizontal bars, columns, and peripheral longitudinal bars indicate the median values, 25th to 75th percentiles, and 10th to 90th percentiles, respectively. OA, on admission; D2, Day 2. n.s., not significant.

Longitudinal changes of coagulofibrinolytic parameters of all patients are depicted in Supplementary Materials Figure S2. Coagulation activation, impairment in anticoagulation, fibrinolytic activation followed by the inhibition of fibrinolysis, and consumption of platelets, fibrinogen, and coagulation factors were observed. We compared the patients’ features, outcomes, and longitudinal changes of coagulofibrinolytic parameters between the two groups according to median SDC-1 value on admission (high SDC-1 group > 99.6 ng/mL, low SDC-1 group ≤ 99.6 ng/mL) (Table 1 and Figure 3a). There were no significant differences in age, sex, and duration from injury to blood sampling between the two groups. Median ISS was significantly higher in the high SDC-1 group than in the low SDC-1 group (27 [15–34] vs. 17 [9–24], p = 0.007). The prevalence of shock and the development of DIC were observed more frequently in the high SDC-1 group compared to the low SDC-1 group (shock, 11 [45.8%] vs. 2 [8.3%], *p* = 0.003; DIC, 10 [41.5%] vs. 1 [4.2%], *p* = 0.002) (Table 1). SDC-1 values on admission in the high and low SDC-1 groups were significantly higher than those in healthy individuals (213.3 [146.6–462.3] ng/mL vs. 24.5 [18.9–47.8] ng/mL, *p* < 0.001; 61.4 [38.5–86.5] ng/mL vs. 24.5 [18.9–47.8] ng/mL, *p* = 0.007). The difference in circulating SDC-1 levels between the two groups was significant until D1 and then gradually declined. Coagulation activity, as measured by TAT and SF, was sustained at higher levels in the high SDC-1 group than in the low SDC-1 group (Figure 3a and Supplementary Materials Figure S3). The natural anticoagulants AT and PC remained at lower levels in the high SDC-1 group than in the low SDC-1 group. Both tPA and tPAI-1, an activator and an inhibitor of fibrinolysis, were significantly higher in the high SDC-1 group than in the low SDC-1 group, but peak levels of tPA and tPAI-1 were attained at different times. Fibrinolytic activity, as shown by PIC, increased immediately after trauma to a greater degree in patients with high SDC-1 levels than in those with low levels, concomitant with consumption of plasminogen (PLG) and antiplasmin assessed by α_2_PI. This initial fibrinolytic activation was shut down at the time of peak tPAI-1 level, with tPA decreasing, and the difference between the two groups disappeared. Higher levels of FDP and D-dimer persisted in the high SDC-1 group than in the low SDC-1 group. Transfusions of platelets, FFP and PRBC were significantly higher in the high SDC-1 group than those in low SDC-1 group (PRBC, 5 [0–14] U vs. 0 [0–6] U, p = 0.033; FFP, 7 [0–16] U vs. 0 [0–0] U, p = 0.005; platelets, 0 [0–0] vs. 0 [0–0], p = 0.020) (Table 1). Consumption of coagulation factors, as shown by Fbg and PT-international normalized ratio (INR), was apparent in the initial phase of trauma (Table 1) but improved over time. Even though we did not detect significant differences in PLT levels, the decreases in values from admission to the nadir on D2 were significantly greater in the high SDC-1 group than in the low SDC-1 group (Figure 3b). Lactate levels were significantly higher in the high SDC-1 group than in the low SDC-1 group (lactate, 38.0 [20.0–52.8] mg/dL vs. 14.0 [9.5–22.5] mg/dL, *p* < 0.001) (Figure 3c), but there were no significant differences in IL-6 values between the two groups.

### 3.4. Association of Circulating SDC-1 with Development of DIC, Prevalence of Shock, and In-Hospital Mortality

**Figure 4:**
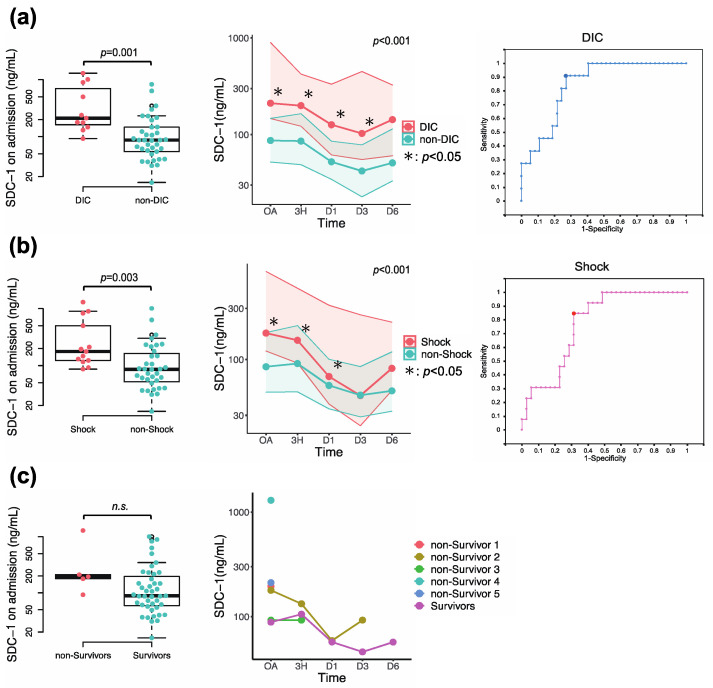
Comparisons of syndecan-1 (SDC-1) levels between (**a**) patients with and without the development of disseminated intravascular coagulation (DIC) (DIC, red dots; non-DIC, green dots), (**b**) patients with and without prevalence of shock (Shock, red dots; non-Shock, green dots), and (**c**) survivors and nonsurvivors (Survivors, green dots; non-Survivors, red dots). Comparisons of SDC-1 levels on admission and those of longitudinal changes between the two groups, and receiver-operating-characteristic (ROC) curve analyses for predicting the development of DIC and the prevalence of shock are represented. Statistical significance of differences in longitudinal changes of SDC-1 values between the two groups was assessed by two-way repeated measures ANOVA (*p* value) with a Bonferroni post hoc test (* *p* < 0.05). The cut-off values were determined by ROC analysis using the Youden index. n.s., not significant.

SDC-1 levels were significantly higher on admission, and higher levels were sustained in patients with DIC compared with those without DIC (SDC-1 on admission, 211.3 [146.8–897.3] ng/mL vs. 86.8 [52.0–147.1] ng/mL, *p* = 0.001), and in patients with shock compared with those without shock (SDC-1 on admission, 211.3 [119.8–665.4] ng/mL vs. 85.5 [49.4–178.7] ng/mL, *p* = 0.003) (Figure 4a,b). ROC curve analyses demonstrated that circulating SDC-1 was a significant predictor of the development of DIC (area under the curve (AUC) = 0.845, cut-off value = 130.38 ng/mL, *p* = 0.001), the prevalence of shock (AUC = 0.774, cut-off value = 116.48 ng/mL, *p* = 0.004) (Figure 4a,b and Table 2). The cumulative development of DIC was significantly higher in the high SDC-1 group than in the low SDC-1 group (*p* = 0.013) (Supplementary Materials Figure S4). No significant differences were observed in SDC-1 values between survivors and nonsurvivors due to the small number of cases (Figure 4c). 

## 4. Discussion

In the present study, we investigated associations between circulating SDC-1 levels, a marker of endothelial glycocalyx degradation, and coagulofibrinolytic responses in the early phase of trauma. Circulating SDC-1 increased immediately after trauma in correlation with coagulofibrinolytic activation. This result indicates that endotheliopathy develops just after trauma, and SDC-1 may be a quantitative index of endotheliopathy which shares a common pathway with trauma-induced coagulopathy. Furthermore, sustained SDC-1 elevation was associated with intense and prolonged coagulation activation, impairment in anticoagulation, and fibrinolytic activation followed by the inhibition of fibrinolysis, which are the primary responses leading to development of DIC in the acute phase of trauma [6,19,20]. An elevated level of circulating SDC-1 was also associated with consumption coagulopathy and the need for transfusion, which consequently revealed the significant association between high SDC-1 levels and development of DIC after trauma. In the current study, patients with DIC sustained higher SDC-1 levels after trauma compared with patients without DIC. These results indicate that endotheliopathy, as evaluated using circulating SDC-1 levels, may be associated with the extent and duration of coagulofibrinolytic responses, which can result in the development of DIC. 

The endothelial glycocalyx maintains vascular homeostasis: that is, regulation of vascular permeability, inhibition of microvascular thrombosis, and regulation of leukocyte adhesion [8,10]. Degradation of the endothelial glycocalyx has been observed in various critical situations, including sepsis [23,24,25,26,27], trauma [12,13,14,16,17,18], malignancy, and cardiovascular diseases, among others [8,9,28]. Degradation of the glycocalyx releases the components such as SDC-1 into the blood stream; thus, circulating SDC-1 may serve as a biomarker of endotheliopathy. Actually, previous reports have demonstrated that SDC-l levels were associated with glycocalyx degradation [29,30] and other markers of endothelial damage such as thrombomodulin [31,32]. Previous studies have shown the association of increased SDC-1 levels with disease severity and poor outcome in trauma patients [12,13,14,15,16,17]. Our study demonstrated that a remarkable elevation in SDC-1 level was observed immediately after trauma, which was significantly associated with the prevalence of shock and the development of DIC. Especially, SDC-1 was sustained at higher levels in patients with DIC than those without DIC in the current study. Few studies in the literature have investigated the chronology of circulating SDC-1 levels; most studies have measured only at a single time point. A previous study of longitudinal changes of SDC-1 in patients with septic shock showed that sustained high SDC-1 levels were closely related to the presence of acute respiratory distress syndrome (ARDS) resulting from the continuous shedding of the endothelial glycocalyx and an impairment of glycocalyx reconstitution [33]. Accordingly, sustained SDC-1 elevation might indicate prolonged vascular injury, which can cause organ dysfunction, including coagulation disorders. Our results suggested that endothelial damage with sustained SDC-1 elevation leading to impairment of glycocalyx reconstitution, as well as the initial damage, can be associated with the development of DIC. Pathogenesis of DIC in trauma is multifactorial such as injury severity, coagulofibrinolytic activation, or shock; therefore, a direct causal relationship between DIC and endotheliopathy, and a threshold effect of SDC-1, have been unsolved in this study. However, our results indicate that SDC-1 levels and coagulofibrinolytic responses share a common pathway after trauma. Furthermore, clearance of SDC-1 is not well understood, but the entire plasma pool is expected to be eliminated by renal excretion within 24 h. However, impaired renal clearance of SDC-1 due to kidney dysfunction may induce several-fold increasing in plasma SDC-1 concentration; thus, further investigation of SDC-1 elimination is needed [34].

TIC is attributed to trauma, in which shock, hypoperfusion, endotheliopathy, and many other factors act synergistically to provoke coagulopathy. TIC manifests a wide range of complications, including bleeding and thrombosis [35,36]. TIC phenotypes change over time, and a dysregulated excessive coagulofibrinolytic response may eventually lead to the development of DIC [6,19,20,35,36]. DIC in the early phase of trauma exhibits an excessive fibrinolytic phenotype, which is characterized by coagulation activation, impairment in anticoagulation, fibrinolytic activation complicated by delayed inhibition of fibrinolysis, and consumption coagulopathy [6,19,20]. In the current study, initial coagulofibrinolytic responses were significantly correlated with elevation in circulating SDC-1 levels. Moreover, coagulation activation (represented by TAT elevation) and impairment in anticoagulation (shown by decreased AT and PC levels) were intense and prolonged in association with increased SDC-1 levels. In the initial phase of trauma, both tPA and tPAI-1 increased in association with high SDC-1 levels, but tPA elevation occurred before PAI-1 elevation. Consequently, fibrinolytic activation by tPA immediately after trauma overcame the inhibition by PAI-1, the levels of which had not yet peaked, resulting in excessive fibrinolytic activation represented by increased PIC levels. This initial response may be compatible with the pathophysiology of DIC with a fibrinolytic phenotype [6,19,20]. Subsequently, PAI-1 achieved peak levels in association with increased SDC-1 levels at which time fibrinolytic activation was drastically inhibited to the extent that differences in SDC-1 levels were not detected. Regarding consumption coagulopathy, there were no significant differences in the time course of PLT and Fbg except for PT-INR. These results may have been influenced by the administration of blood transfusion for consumption coagulopathy, which depended on PLT and fibrinogen Fbg levels. Actually, the transfusions of platelets, FFP, and PRBC were significantly higher in the high SDC-1 group compared to the low SDC-1 group. This finding indirectly suggests that consumptive coagulopathy was more pronounced in patients with high SDC-1 levels. Overall, endothelial glycocalyx degradation, represented by circulating SDC-1 levels, was associated with intense and prolonged coagulofibrinolytic responses after trauma and the development of DIC. 

The endothelium plays a pivotal role in hemostasis and prevention of coagulation. The endothelial glycocalyx contains heparan sulfate proteoglycan and provides anticoagulant molecules such as antithrombin and thrombomodulin, thus preventing platelet aggregation and coagulation. Moreover, the glycocalyx regulates excessive shear stress, which can cause platelet activation via dysregulated secretion of von Willebrand factor [8,23,37]. Recent studies of sepsis have demonstrated an association between SDC-1 levels and the development of DIC [24,38]. TIC has been reported to be associated with SDC-1 elevation [18,39], but the relationship between development of DIC and SDC-1 levels is not fully understood. TIC is distinct from other coagulopathies such as sepsis-induced coagulopathy, but dysregulated coagulofibrinolytic response induces progression to the final pathway of DIC [35,36]. Therefore, TIC and DIC in the early phase of trauma show the similar coagulofibrinolytic responses [19]. Johansson PI et al. reported that progression to overt DIC may occur several hours after trauma [39]. Thus, in the current study, initial assessment was performed at a median of 60 min after injury, and DIC was generally diagnosed several hours after admission. However, our results clearly demonstrated that the coagulofibrinolytic responses that can cause DIC occurred just after trauma. Furthermore, circulating SDC-1 levels were elevated in the initial phase of trauma, which was correlated with these coagulofibrinolytic responses, and elevated SDC-1 levels were sustained prominently in patients with DIC. Thereby, early intervention for these excessive coagulofibrinolytic responses and endotheliopathy should be considered to avoid progression to DIC. Regarding consumption coagulopathy, sufficient transfusion to maintain hemostasis is essential and has been usually performed. In addition, we should simultaneously manage the endotheliopathy as one of the therapeutic targets for TIC from the early phase of trauma, but an early diagnostic, therapeutic, and protective method for glycocalyx damage has not been established yet. In some previous studies, excessive fluid resuscitation has been reported to cause glycocalyx degradation [40,41]. Transfusion of fresh frozen plasma has been proposed to be glycocalyx protective [42]. Moreover, coagulofibrinolytic markers may not be indicative of the functional contributions of coagulofibrinolytic status as they evaluate isolated portions of the coagulation cascade. Viscoelastic hemostatic assays (VHAs) are available as whole blood point of care tests for a global assessment of coagulation and fibrinolysis and have been used for the management of major bleeding and guiding transfusion therapy [43,44]. Larger scale studies to confirm the findings of the current study with the addition of the evaluation of VHA parameters are warranted to establish effective strategies for endotheliopathy of trauma.

Several limitations of the present study should be noted. This was a retrospective, single-center study with a small sample size and thus does not allow for independent evaluation of cause and effect relationships. Second, 5 nonsurvivors of 48 patients were not followed until 7 days after admission, which may have distorted the results. Larger-scale studies are needed to test the hypothesis that has arisen from the present study. Third, TIC is complex and heterogeneous, and the prevalence of shock, therapeutic intervention, and trauma mechanism can further increase the heterogeneity of this study. A large-scale study adjusted for factors associated with TIC and endotheliopathy of trauma is needed to confirm these results.

## 5. Conclusions

Increased circulating levels of syndecan-1 were associated with excessive responses in coagulofibrinolysis over time, such as coagulation activation, impairment of anticoagulation, fibrinolytic activation, and consumption coagulopathy after trauma. Endotheliopathy represented by SDC-1 elevation was associated with trauma-induced coagulopathy, which can cause the development of DIC. Larger scale studies to confirm the findings of the current study with the addition of the evaluation of VHA parameters are warranted.

## Figures and Tables

**Table 1:** Patients clinical features and outcomes.

		ALL (n = 48)	SDC-1 on day 0		
			>99.6 ng/mL (n = 24)	≤99.6 ng/mL (n = 24)	*p* Value
Patient characteristics					
Age	years	61.5 (42.0–72.6)	67.0 (42.3–75.3)	55.0 (41.3–71.0)	0.672
Sex; male/female	n, (%)	37 (77.1)/11 (22.9)	16 (66.7)/8 (33.3)	21 (87.5)/3 (12.5)	0.227
Injury Severity Score (ISS)		19 (13–29)	27 (15–34)	17 (9–24)	0.007
shock	n, (%)	13 (27.1)	11 (45.8)	2 (8.3)	0.003
ISTH-overt DIC (+)	n, (%)	11 (22.9)	10 (41.7)	1 (4.2)	0.002
Timing of DIC diagnosis n, (%)					
OA		2 (4.2)	2 (8.3)	0 (0.0)	0.149
OA–3H		7 (14.6)	6 (25.0)	1 (4.2)	0.041
3H–24H		2 (4.2)	2 (8.3)	0 (0.0)	0.149
24H–		0 (0.0)	0 (0.0)	0 (0.0)	NA
Duration from injury to blood sampling					
	minutes	60 (43–110)	62 (36–118)	58 (46–82)	1.000
Laboratory data [normal range]					
HGB [11.3–15.2]	g/dL	13.1 (11.6–14.0)	13.0 (10.8–13.7)	13.3 (11.9–15.0)	0.095
HCT [34.3–45.2]	%	38.2 (34.2–41.3)	38.2 (31.7–40.1)	38.7 (35.3–43.0)	0.111
PLT [13.1–36.9]	×10^4^/μL	21.8 (17.9–26.8)	22.3 (18.2–26.8)	21.4 (17.7–26.7)	0.703
PT-INR [0.85–1.15]		0.97 (0.92–1.08)	1.03 (0.95–1.21)	0.93 (0.92–1.00)	0.018
APTT [21.5–43.1]	sec	23.9 (21.7–28.1)	26.4 (21.9–30.5)	23.0 (21.7–25.0)	0.039
Fbg [200–400]	mg/dL	223 (184–270)	206 (178–246)	246 (197–294)	0.036
FDP [<5.0]	μg/mL	89.4 (27.3–164.3)	154.3 (64.2–226.1)	39.9 (16.6–96.9)	<0.001
D-dimer [<1.0]	μg/mL	46.1 (14.7–82.5)	78.0 (33.9–122.2)	20.5 (8.4–49.9)	<0.001
TAT [<3.0]	μg/L	115.3 (49.3–200.0)	194.2 (127.0–235.4)	60.1 (41.4–114.1)	<0.001
PIC [0.0–0.8]	μg/mL	10.4 (4.8–27.9)	16.5 (8.8–37.8)	5.2 (3.3–10.8)	0.001
tPA [1270–8840]	pg/mL	6468 (4208–10,411)	9696 (5638–15,685)	5863 (3287–8075)	0.006
tPAI-1 [<50]	ng/mL	31 (15–63)	46 (28–81)	21 (11–57)	0.010
AT [80.0–120.0]	%	88.7 (77.4–97.1)	82.4 (66.8–93.3)	92.5 (87.9–100.5)	0.003
PC [82.0–112.0]	%	82.6 (71.3–108.4)	75.0 (63.4–97.2)	87.8 (74.5–111.6)	0.039
α2PI [80.0–130.0]	%	83.6 (67.6–100.3)	74.3 (58.8–88.1)	96.9 (80.3–106.8)	0.001
PLG [80.0–130.0]	%	91.2 (75.7–103.2)	82.8 (68.9–96.5)	98.5 (86.5–106.7)	0.028
IL-6 [<4.0]	pg/mL	122 (33–277)	177 (87–395)	63 (22–262)	0.097
Lactate [3.3–14.9]	mg/dL	22.0 (14.0–42.5)	38.0 (20.0–52.8)	14.0 (9.5–22.5)	<0.001
Alb [3.9–4.9]	g/dL	3.9 (3.6–4.2)	3.7 (3.3–4.0)	4.1 (3.7–4.2)	0.044
BUN [7–21]	mg/dL	17 (15–21)	18 (14–23)	17 (15–21)	0.535
Cre [0.65–1.07]	mg/dL	0.88 (0.75–1.07)	0.98 (0.78–1.12)	0.87 (0.73–0.98)	0.166
SDC-1	ng/mL	99.6 (61.1–214.3)	213.3 (146.6–462.3)	61.4 (38.5–86.5)	<0.001
Transfusion					
PRBC	Units	1 (0–10)	5 (0–14)	0 (0–6)	0.033
FFP	Units	0 (0–8)	7 (0–16)	0 (0–0)	0.005
Platelets	Units	0 (0–0)	0 (0–0)	0 (0–0)	0.020
Intervention					
Craniotomy	n, (%)	2 (4.2)	2 (8.3)	0 (0.0)	0.149
Thoracotomy	n, (%)	2 (4.2)	1 (4.2)	1 (4.2)	1.000
Laparotomy	n, (%)	3 (7.1)	2 (8.3)	1 (4.2)	0.551
IVR	n, (%)	10 (23.8)	8 (33.3)	2 (8.3)	0.033
ORIF	n, (%)	20 (47.6)	8 (33.3)	12 (50.0)	0.242
Outcome					
In-hospital mortality	n, (%)	5 (10.4)	4 (16.7)	1 (4.2)	0.156

NA, not applicable; OA, on admission; 3H, 3 h after admission; 24H, 24 h after admission; ISTH, the International Society on Thrombosis and Haemostasis; DIC, disseminated intravascular coagulation; HGB, hemoglobin; HCT, hematocrit; PLT, platelet count; PT-INR, prothrombin time–international normalized ratio; APTT, activated partial thromboplastin time; Fbg, fibrinogen; FDP, fibrin/fibrinogen degradation product; TAT, thrombin–antithrombin complex; PIC, plasmin-α_2_-plasmin inhibitor complex; tPA, tissue plasminogen activator; tPAI-1, total plasminogen activator inhibitor-1; AT, antithrombin; PC, protein C; α_2_PI, α_2_-plasmin inhibitor; PLG, plasminogen; IL-6, Interleukin-6; Alb, albumin; BUN, blood urea nitrogen; Cre, creatinine; SDC-1, syndecan-1; PRBC, packed red blood cell; FFP, fresh-frozen plasma; IVR, interventional radiology; ORIF, open reduction and internal fixation.

**Table 2:** Cut-off values, sensitivity, and specificity of syndecan-1 (SDC-1) for predicting the development of ISTH overt-DIC and the prevalence of shock.

	Cut-Off Value	Sensitivity (%)	Specificity (%)	AUC	Standard Error	*p* Value	95%CI	
							Lower Limit	Upper Limit
DIC	130.38 ng/mL	90.9	73.0	0.845	0.057	0.001	0.734	0.957
Shock	116.48 ng/mL	84.6	68.6	0.774	0.067	0.004	0.643	0.905

AUC, area under the curve; CI, confidence interval.

## Data Availability

The datasets for the current study are available from the corresponding author upon reasonable request.

## Supplementary Materials

The following supporting information can be downloaded at: https://www.mdpi.com/article/10.3390/jcm12134386/s1, Figure S1: Pearson’s correlation matrix among coagulofibrinolysis-related markers including syndecan-1 (SDC-1). Figure S2: Time course changes in coagulofibrinolytic markers including syndecan-1 (SDC-1). Figure S3: Comparisons of the distribution of soluble fibrin (SF) levels on admission (OA), 3 h after admission (3H), and on day 1, 2, 3 and 6 (D1, D2, D3 and D6) based on the median syndecan-1 (SDC-1) level on admission. Figure S4: A comparison of the cumulative development of DIC between High and Low SDC-1 groups.

## Abbreviations

Alb: albumin; APTT: activated partial thromboplastin time; AT: antithrombin; DIC: disseminated intravascular coagulation; Fbg: fibrinogen; FDP: fibrin/fibrinogen degradation product; FFP: fresh-frozen plasma; HCT: hematocrit; HGB: hemoglobin; ISS: Injury Severity Score; ISTH: the International Society on Thrombosis and Haemostasis; IVR: interventional radiology; ORIF: open reduction and internal fixation; PC: protein C; PIC: plasmin-α_2_-plasmin inhibitor complex; PLG: plasminogen; PRBC: packed red blood cell; PT-INR: prothrombin time–international normalized ratio; SDC-1: syndecan-1; SF: soluble fibrin; TAT: thrombin–antithrombin complex; TIC: trauma-induced coagulopathy; tPA: tissue plasminogen activator; tPAI-1: total plasminogen activator inhibitor-1; α_2_PI: α_2_-plasmin inhibitor.
