# Supplementary material for: Circulating Syndecan-1 Levels Are Associated with Chronological Coagulofibrinolytic Responses and the Development of Disseminated Intravascular Coagulation (DIC) after Trauma: A Retrospective Observational Study
Source: J Clin Med. 2023 Jun 29;12(13):4386. doi: 10.3390/jcm12134386 (PMC10342599; doi:10.3390/jcm12134386)
Supplement: Supplementary file 1 [file jcm-12-04386-s001.zip › jcm-2460028-supplementary.pdf]

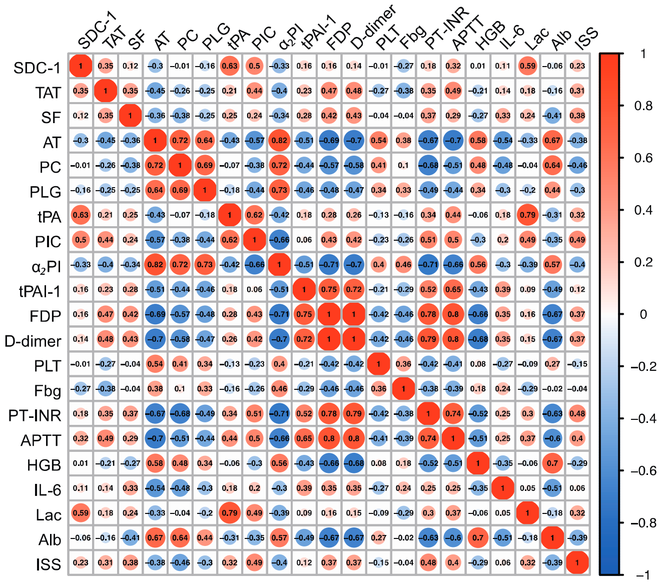

Supplementary Figure S1. Pearson's correlation matrix among coagulofibrinolysis-related markers including syndecan-1 (SDC-1).

Correlation coefficients are colored from 1 (red) to −1 (blue) and shaded according to strength of correlation.

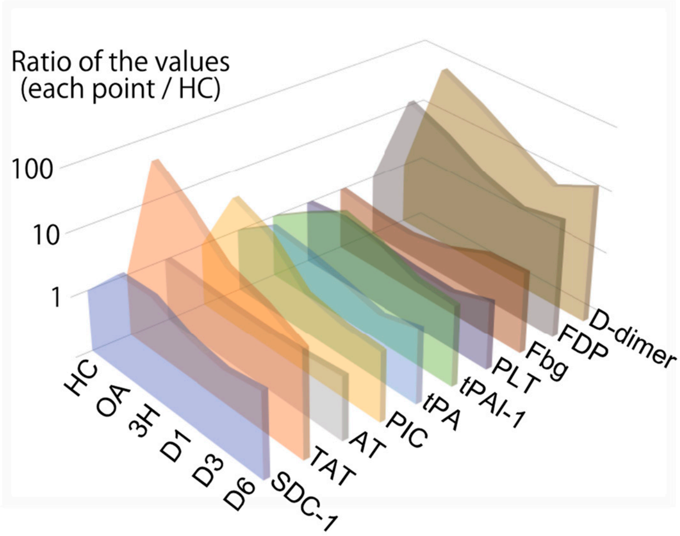

Supplementary Figure S2. Time course changes in coagulofibrinolytic markers including syndecan-1 (SDC-1).

The vertical axis shows the ratio of the median values of the trauma patients on admission (OA), 3 hours after admission (3H), and on days 1, 3 and 6 (D1, D3, and D6) to the median values of healthy control (HC) samples.

TAT, thrombin-antithrombin complex; AT, antithrombin; PIC, plasmin-α_2-plasmin inhibitor complex; tPA, tissue plasminogen activator; tPAI-1, total plasminogen activator inhibitor-1; PLT, platelet; Fbg, fibrinogen; FDP, fibrin/fibrinogen degradation product.

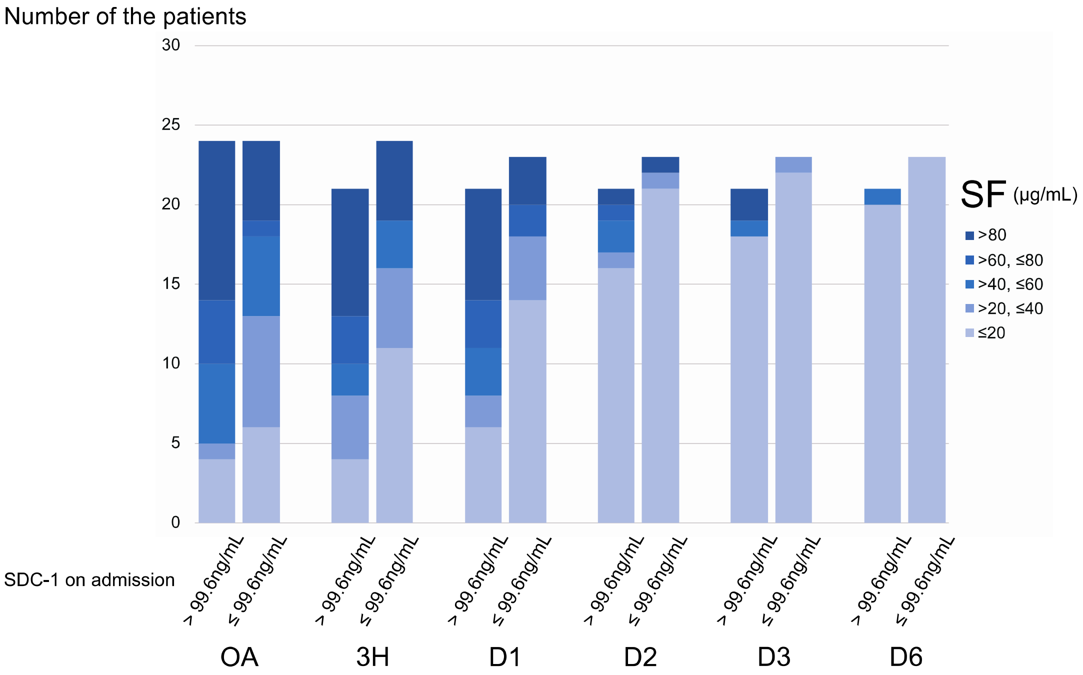

Supplementary Figure S3. Comparisons of the distribution of soluble fibrin (SF) levels on admission (OA), 3 hours after admission (3H), and on day 1, 2, 3 and 6 (D1, D2, D3 and D6) based on the median syndecan-1 (SDC-1) level on admission.

The vertical axis shows the number of the patients.

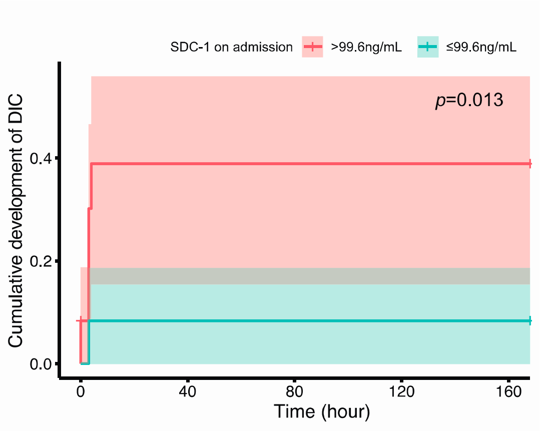

Supplementary Figure S4. A comparison of the cumulative development of DIC between High and Low SDC-1 groups.

The red line represents the values of the patients with SDC-1 level on admission >99.6ng/mL, and the green line represents those with SDC-1 level on admission ≤ 99.6ng/mL.
